# Can Biomarkers Identify Women at Increased Stroke Risk? The Women's Health Initiative Hormone Trials

**DOI:** 10.1371/journal.pctr.0020028

**Published:** 2007-06-15

**Authors:** Charles Kooperberg, Mary Cushman, Judith Hsia, Jennifer G Robinson, Aaron K Aragaki, John K Lynch, Alison E Baird, Karen C Johnson, Lewis H Kuller, Shirley A. A Beresford, Beatriz Rodriguez

**Affiliations:** 1 Division of Public Health Sciences, Fred Hutchinson Cancer Research Center, Seattle, Washington, United States of America; 2 College of Medicine, University of Vermont, Burlington, Vermont, United States of America; 3 Department of Medicine, George Washington University, Washington, District of Columbia, United States of America; 4 Department of Medicine, University of Iowa, Iowa City, Iowa, United States of America; 5 Stroke Neuroscience Unit, National Institute of Neurological Disorders and Stroke, Bethesda, Maryland, United States of America; 6 Department of Preventive Medicine, University of Tennessee Health Sciences Center, Memphis, Tennessee, United States of America; 7 Department of Epidemiology, University of Pittsburgh School of Public Health, Pittsburgh, Pennsylvania, United States of America; 8 Department of Epidemiology, School of Public Health and Community Medicine, University of Washington, Seattle, Washington, United States of America; 9 John A. Burns School of Medicine, University of Hawaii at Manoa, Honolulu, Hawaii, United States of America

## Abstract

**Objective:**

The Women's Health Initiative hormone trials identified a 44% increase in ischemic stroke risk with combination estrogen plus progestin and a 39% increase with estrogen alone. We undertook a case-control biomarker study to elucidate underlying mechanisms, and to potentially identify women who would be at lower or higher risk for stroke with postmenopausal hormone therapy (HT).

**Design:**

The hormone trials were randomized, double-blind, and placebo controlled.

**Setting:**

The Women's Health Initiative trials were conducted at 40 clinical centers in the United States.

**Participants:**

The trials enrolled 27,347 postmenopausal women, aged 50–79 y.

**Interventions:**

We randomized 16,608 women with intact uterus to conjugated estrogens 0.625 mg with medroxyprogesterone acetate 2.5 mg daily or placebo, and 10,739 women with prior hysterectomy to conjugated estrogens 0.625 mg daily or placebo.

**Outcome Measures:**

Stroke was ascertained during 5.6 y of follow-up in the estrogen plus progestin trial and 6.8 y of follow-up in the estrogen alone trial.

**Results:**

No baseline clinical characteristics, including gene polymorphisms, identified women for whom the stroke risk from HT was higher. Paradoxically, women with higher baseline levels of some stroke-associated biomarkers had a lower risk of stroke when assigned to estrogen plus progestin compared to placebo. For example, those with higher IL-6 were not at increased stroke risk when assigned to estrogen plus progestin (odds ratio 1.28) but were when assigned to placebo (odds ratio 3.47; *p* for difference = 0.02). Similar findings occurred for high baseline PAP, leukocyte count, and D-dimer. However, only an interaction of D-dimer during follow-up interaction with HT and stroke was marginally significant (*p* = 0.03).

**Conclusions:**

Biomarkers did not identify women at higher stroke risk with postmenopausal HT. Some biomarkers appeared to identify women at lower stroke risk with estrogen plus progestin, but these findings may be due to chance.

## Editorial Commentary

**Background:** The Women's Health Initiative hormone trials originally set out to evaluate whether postmenopausal hormone therapy (HT, estrogen in the case of women who had had a hysterectomy, and estrogen plus progestin for women who had not had a hysterectomy) reduced the risk of heart attacks and strokes, as compared to placebo. The trials were stopped early, and the investigators found that both estrogen alone, as well as estrogen plus progestin increased the risk of stroke amongst women participating in the trials. As part of a secondary analysis of data from these trials, the investigators aimed to explore possible associations between various biological markers (such as variants in particular genes, and levels of particular lipids, proteins, and other markers in blood), and the risk of a woman experiencing a stroke in the trials. Specifically, they wanted to evaluate whether there was any evidence for particular markers being associated with the risk of a stroke; and then whether that risk was modified by whether a woman took HT in the trials.

**What the trial shows:** In this study, the researchers collected early cases of ischemic stroke in the trials (combining cases among women taking estrogen with those for women taking both estrogen and progestin), and matched these to control individuals, or women participating in the trials who did not experience a stroke. Two hundred five women who experienced a stroke were compared to 878 control individuals. The markers analyzed included those for which there was already some evidence for an association with stroke. Several clinical characteristics and some biomarkers, as measured at the start of the trial, but none of the gene variants, were linked with later risk of stroke. However, none of these clinical characteristics or gene variants specifically identified women who were at greater risk of experiencing a stroke within the HT arms of the trial. High levels of two biomarkers, IL-6 and PAP, did seem to identify women who were at lower risk of experiencing a stroke within the HT arms of the trial. This finding is interesting, because high levels of these markers had previously been suggested as being associated with a higher risk of stroke. Levels of several biomarkers changed during the trial, but for only one biomarker, D-dimer, did the change (an increase in levels) seem to predict higher risk of stroke amongst women receiving HT.

**Strengths and limitations:** A particular strength of this study includes the nesting of a case-control study within the Women's Health Initiative trials, in which HT or placebo was randomly assigned. This design minimizes the chance that individuals taking HT differ in their stroke risk from individuals taking placebo. However, the power of this study to detect anything other than large associations is limited; together with the limitation of multiple statistical testing, the findings here must be interpreted as hypotheses for further study and not definitive conclusions.

**Contribution to the evidence:** This study adds data relating to possible predictive risk markers for stroke among users of HT. The hypotheses raised here remain to be tested in further studies.

## INTRODUCTION

The Women's Health Initiative hormone trials were designed to evaluate the role of postmenopausal hormone therapy (HT) in cardiovascular risk reduction. Unexpectedly, both estrogen with progestin and estrogen alone increased stroke risk [1,2]. Evaluation of clinical characteristics and a limited number of biomarkers in the individual trials [3,4] failed to identify women at higher or lower risk for stroke with HT.

In this analysis, we pooled stroke outcomes from the Women's Health Initiative hormone trials to evaluate a broad range of genetic and baseline phenotypic biomarkers in order to formulate hypotheses about how HT increased stroke risk. We evaluated the association between baseline biomarkers and stroke, whether that association was modified by HT, whether biomarkers were influenced by HT, and whether biomarker changes influenced stroke risk.

The biomarkers analyzed in this paper were part of a slightly larger panel of markers that were thought by members of the Women's Health Initiative laboratory working group to be associated with either stroke, venous thrombotic disease, or myocardial infarction (MI). Before analyzing the data we restricted our attention to those markers for which we felt there was evidence for an association with stroke, or for a modification of the effects on stroke in the presence of HT. Other markers in the original panel were not analyzed in relation to stroke. The major focus of this biomarker study was to understand why HT increases stroke risk.

## METHODS

**Figure 1:**
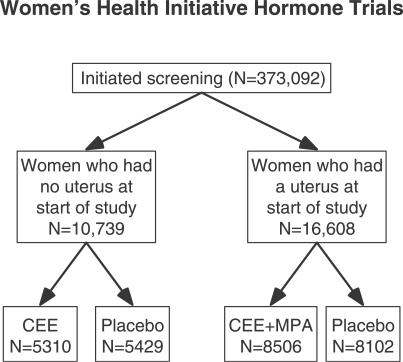
Women's Health Initiative Hormone Trials CEE, conjugated equine estrogens; MPA, medroxyprogestrone acetate.

Details of the design, recruitment, randomization, data collection, intervention, and outcomes ascertainment procedures of the Women's Health Initiative hormone trials, including CONSORT diagrams, have been published [1,2,5–8]. Also see Figure 1.

### Participants and Interventions

Between November 1993 and October 1998, postmenopausal women with prior hysterectomy (*n* = 10,739) were randomized to conjugated equine estrogens 0.625 mg/day (Premarin, Wyeth Pharmaceticals, http://www.wyeth.com) or placebo; those with an intact uterus (*n* = 16,608) were randomized to conjugated equine estrogens 0.625 mg/day with medroxyprogestrone acetate 2.5 mg/day (Prempro, Wyeth Pharmaceticals) or placebo (Figure 1). Participants provided informed consent in a form approved by local institutional review boards. The estrogen plus progestin arm was stopped after 5.6 y of follow-up upon recommendation of the Data and Safety Monitoring Board because of increased breast cancer risk [1]; the estrogen alone trial was stopped after 6.8 y of follow-up by the National Institutes of Health because of increased stroke risk and lack of cardioprotection [2].

Because of early adverse effects of HT on cardiovascular events in the Women's Health Initiative a nested case-control study was carried out. Participants with stroke, MI, and venous thromboembolism (VTE) as of February 2001 were matched to controls on age, randomization date, hysterectomy status, and prevalent cerebrovascular disease. For this paper we analyzed the cases of ischemic stroke, and the combined controls for each of the three outcomes to increase the power of detecting an association. In particular, this study included 205 participants who had ischemic strokes cases and 878 controls. Of the participants who had a cases stroke, 11 also experienced an MI and seven also experienced a VTE event by February 2001. Manuscripts analyzing the biomarkers discussed in this paper in relation to VTE and MI are in preparation. Participants provided informed consent using forms approved by local institutional review boards.

### Outcomes

#### Follow-up and end-point determination.

Clinical outcomes were identified by semiannual questionnaires and classified by centrally-trained local adjudicators following medical record review [8]. All locally adjudicated stroke cases, and self-reported strokes not validated by local adjudicators, were centrally-adjudicated by stroke neurologists. All adjudicators were blinded to treatment assignment. Ischemic strokes were classified according to the Trial of Org 10172 in Acute Stroke Therapy (TOAST) and Oxfordshire subtype classifications. The TOAST stroke subtypes were as follows: large artery artherosclerosis (9%), cardioembolic (12%), small vessel occlusions (29%), other etiology (5%), and unknown etiology (45%). The Oxfordshire classifications were as follows: total anterior infarct (7%), partial anterior circulation infarct (40%), lacunar infarction (37%), and posterior circulation infarct (17%).

#### Genetic and biomarker analysis.

Blood samples were collected from all participants at baseline and 1 y. The baseline blood samples were analyzed for all 205 participants who experienced a stroke and 878 controls; the year 1 blood was analyzed for the 138 participants who experienced their stroke after the year 1 blood collection, and the 603 controls who were matched to a participant who experiences her stroke, MI, or VTE event after the year 1 blood collection.

Lipid profiles were analyzed in EDTA-treated plasma. High-density lipoprotein (HDL) was precipitated with heparin manganese (Dade-Behring, http://www.dadebehring.com). interleukin-6 (IL-6, ultra-sensitive ELISA, R&D Systems, http://www.rndsystems.com), E-selectin, and matrix metalloproteinase-9 (MMP-9) were measured at Medical Research Laboratories (http://www.mrli.ppdi.com). C-reactive protein (N-High Sensitivity CRP, Dade-Behring,), fibrinogen (clot rate assay: Diagnostica Stago, http://www.stago-us.com), factor VIII activity (clotting time on mixing with factor VIII deficient plasma using STA-Deficient VIII; Diagnostica Stago), von Willebrand factor activity and fibrin D-dimer (immunoturbidometric assays: Liatest von Willebrand factor, Liatest D-Di; Diagnostica Stago), plasminogen activator inhibitor-1 antigen (PAI-1) and plasmin-antiplasmin complex (PAP, by in house immunoassay [9,10]), prothrombin fragment 1.2 (ELISA, Dade-Behring), and thrombin activatable fibrinolysis inhibitor (TAFI; immunoassay with antibodies from Affinity Biologicals, http://www.affinitybiologicals.com) were measured at the Laboratory for Clinical Biochemistry Research, University of Vermont (http://www.med.uvm.edu/lcbr/HP-DEPT.ASP?SiteAreaID=513). Complete blood counts were performed in clinics' local laboratories. Genetic polymorphisms were assayed at Wake Forest University (http://www.wfu.com); we assayed estrogen receptor 2-A1730G (NCBI SNP identification rs4986938), glycoprotein 1bα-M145T (rs6065), glycoprotein IIIa–P1 (rs5918), and the Leiden University (Factor V Leiden, thermolabile variant of MTHF, PAI-1 4G/5G). No other biomarkers were analyzed for the participants in this study.

### Statistical Methods

All baseline marker values were log-transformed due to skewed distributions and for consistency; differences from baseline to year 1 were analyzed on the original scale. Logistic regression models were controlled for age and study (estrogen alone or estrogen plus progestin), race, BMI, waist-hip ratio, smoking, alcohol consumption, physical activity, diabetes mellitus, prevalent coronary disease (including six cases of atrial fibrillation), prevalent cerebrovascular disease, blood pressure (BP), use of antihypertension medication, aspirin, and statins at baseline. Analyses involving year 1 biomarker data only involved 138 of 205 participants who experienced their stroke after that analysis, and 603 (of 878) matched controls.

We assessed the appropriateness of using biomarkers log linearly in generalized additive models [11] using stroke as response, correcting for risk factors; linearity was rejected for CRP, TAFI, and platelets. For those markers we examined both linear and quadratic models. While we used markers linearly to assess significance (the more powerful analysis), we do not report the coefficients in the logistic regression model, but rather the more easily interpreted odds ratios (OR) comparing quartiles or quintiles. Thus, there is no one-to-one correspondence between *p*-values for models below 0.05 and confidence intervals for OR not containing 1.

Sensitivity analyses that excluded potential outliers were carried out; except where noted, results were unaffected. All analyses involving TAFI were repeated comparing the subjects with TAFI values above the 90th percentile (7.4 μg/ml) with those below the 25th percentile, as we a priori hypothesized that only very high TAFI levels influence stroke outcome. We only refer to this additional analysis where it differs from the analysis of TAFI on a log linear scale. As most strokes that were analyzed in this study happened early in the trials, most subjects were adhering to study medication. Of the 205 incident stroke cases in this study, only 32 out of 118 of the subjects on HT and 16 out of 87 subjects on placebo were nonadherent 6 mo before their stroke.

## RESULTS

### Baseline Data

Baseline characteristics are shown by case-control status (Table 1). As this study only included participants who had strokes before February 2001, the OR of stroke for active treatment (unadjusted OR 1.53, 95% confidence interval [CI] 1.01–2.32; adjusted for confounders OR 1.51, 95% CI 0.95–2.38) was comparable to the full duration of the trials [3,4].

**Table 1:**
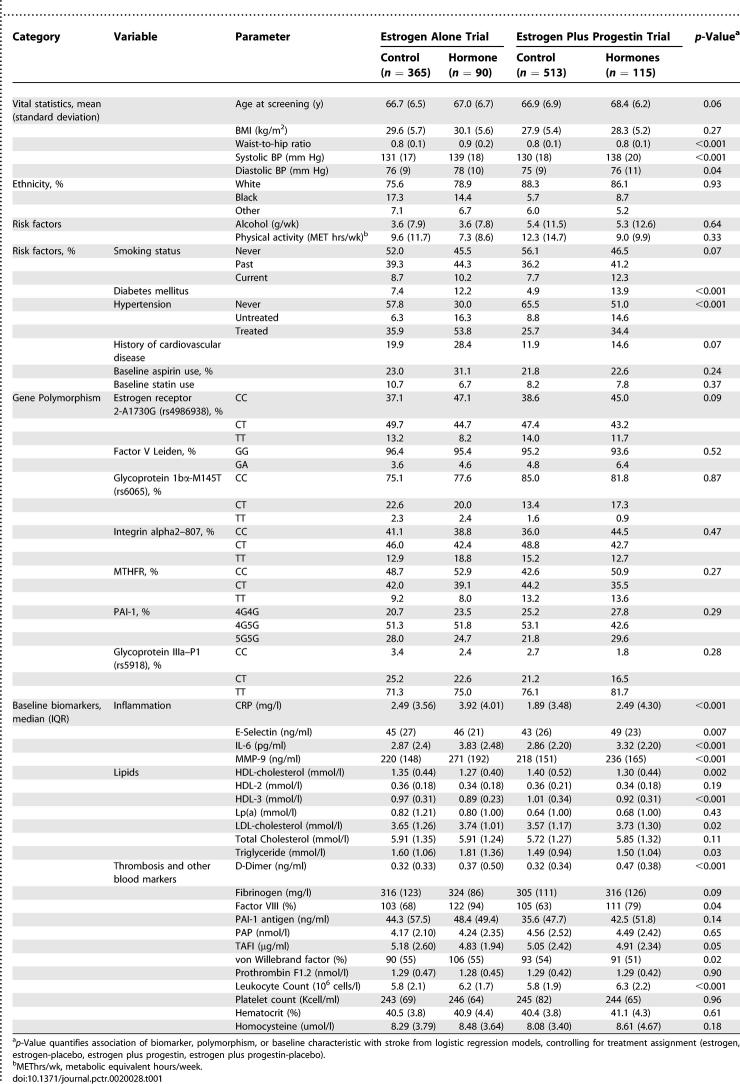
Baseline Characteristics by Case-Control Status and Hormone Trial

Of the 14 clinical characteristics, five were associated with stroke (about one would be expected by chance); of the 23 biomarkers, 13 were associated with stroke (one or two would be expected by chance); none of the seven polymorphisms was associated with stroke. Among controls, Hardy-Weinberg equilibrium was not rejected for any of the polymorphisms. For all biomarkers we fitted a model including an interaction between biomarker and trial enrollment (estrogen alone or estrogen plus progestin versus placebo). Of 30 biomarkers and polymorphisms, only the interaction between hematocrit and trial was marginally significant (*p =* 0.07). As one or two significant results would be expected by chance, this suggests that there were no interaction effects between the trial (estrogen plus progestin or estrogen alone) and the biomarkers. This result, together with the fact that the overall effects of HT on stroke were similar in both trials, suggests that it was appropriate to combine the estrogen plus progestin trial and the estrogen alone trial for analyses of the effects of these biomarkers on stroke.

### Outcomes and Estimation

#### Risk of ischemic stroke for biomarkers.

For each baseline biomarker the stroke risk was compared in multivariate analyses (Table 2). For continuous biomarkers we used log-linear models, and for the polymorphisms we used additive genetic models. As continuous variables, IL-6 (*p* = 0.02), CRP (*p* = 0.03), MMP-9 (*p* = 0.004), D-dimer (*p* < 0.001), LDL (*p* = 0.03), von Willebrand factor (*p* = 0.10), and factor VIII activity (*p* = 0.10) were positively associated with stroke, whereas HDL-3 was inversely associated (*p* = 0.02) with stroke. Of the 30 biomarkers analyzed, seven had a statistically significant association with stroke at the level of *p* = 0.05; one or two markers would be expected to be significant by chance. While TAFI was not strongly associated with stroke when analyzed as a continuous variable (*p* = 0.09), the OR of stroke for TAFI above the 90th percentile compared to the lowest quartile was 0.24 (95% CI 0.09–0.59; *p* = 0.003). Several other biomarkers showed less significant associations.

**Table 2:**
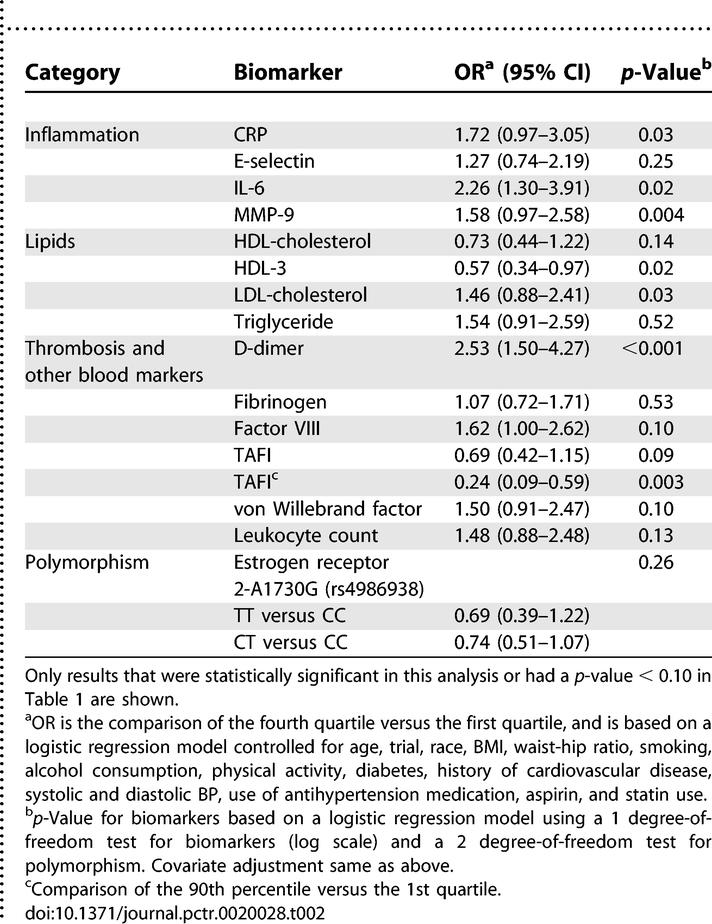
Risk of Ischemic Stroke for Baseline Biomarkers: Highest Quartile versus Lowest Quartile

#### Risk of ischemic stroke with baseline biomarkers by treatment assignment.

Of the 30 biomarkers studied, the *p*-value for interaction between biomarker level and treatment assignment in the pooled cohorts nearly reached significance for IL-6 (*p* = 0.06) and was significant for PAP (*p* = 0.02) (Table 3). As one or two statistically significant results are expected by chance, these results may be due to chance. For these markers, as well as D-dimer and the leukocyte count, the pattern for the estrogen plus progestin trial suggested a smaller OR associated with elevated biomarkers for the active arm than the placebo arm of the trial; no such pattern was observed in the estrogen alone trial. A post-hoc analysis restricted to the estrogen plus progestin trial yielded the following *p*-values for an interaction between baseline biomarker and HT with stroke in the estrogen plus progestin trial: IL-6 (*p =* 0.02), PAP (*p =* 0.002), D-dimer (*p =* 0.02), and leukocyte count (*p =* 0.04), suggesting that individuals with high levels of these biomarkers at baseline have lower stroke risk with estrogen plus progestin than women with lower levels of these biomarkers.

**Table 3:**
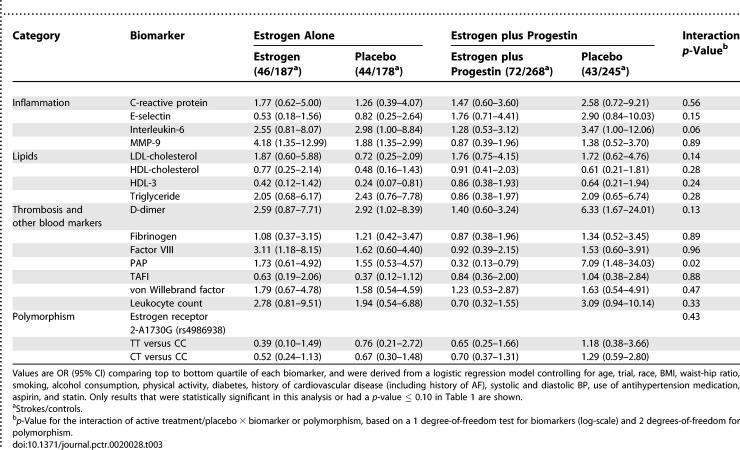
Ischemic Stroke Risk with Baseline Biomarker by Treatment Assignment

We did not find differences in biomarker by treatment assignment between stroke subtypes. In particular, for these results we found no significant differences between ischemic strokes being classified as large artery atherosclerosis or cardioembolism and those classified as small vessel occlusion (unpublished data).

#### Biomarker change from baseline to year 1.

One-year changes in biomarkers are shown in Table 4. Several inflammatory (*p <* 0.001 for CRP, E-selectin, and MMP-9) and thrombotic biomarkers (*p <* 0.001 for PAI-1 and PAP) were altered by HT, as were lipids (all *p <* 0.001). When we removed five extreme changes in D-dimer, the *p*-value for that marker changed from 0.02 to 0.005. No other outliers had a substantial effect. Overall, 14 of the 20 biomarkers had significant changes from baseline to year 1, far more than the one that would be expected by chance.

**Table 4:**
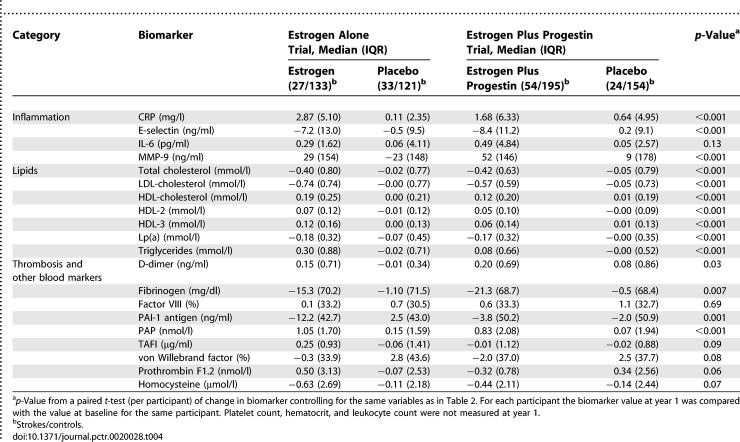
Changes in Biomarkers from Baseline to Year 1

#### Risk of ischemic stroke by change in biomarker level from baseline to year 1.

One-year changes from baseline values were associated with stroke risk in multivariate analysis for only one biomarker: von Willebrand factor (lowest quintile of change OR 0.66, 95% CI 0.35–1.23; middle quintile, including no change, referent; highest quintile OR 0.95, 95% CI 0.51–1.74; *p =* 0.04).

#### Changes in biomarkers and the association of HT with stroke.

Of the 20 biomarkers measured at baseline and 1 y, only the change in D-dimer demonstrated an interaction with randomization assignment (*p =* 0.03, Table 5). For women whose D-dimer increased, the risk of stroke was higher with HT (estrogen plus progestin and estrogen alone combined, OR 1.38 for the top quintile of change) while for women whose D-dimer decreased, the risk of stroke was lower (OR 0.49 for the bottom quintile).

**Table 5:**
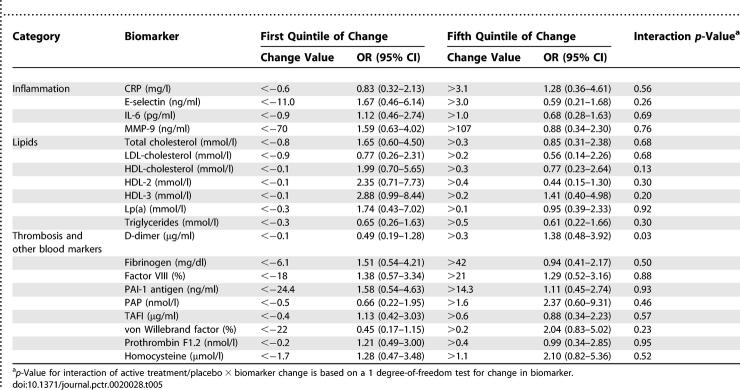
Ischemic Stroke Risk by Treatment Assignment Modified by Change in Biomarker, Baseline to Year 1

We examined whether changes in biomarker level could be considered an intermediate outcome on the pathway from HT to ischemic stroke, in that inclusion of a biomarker change in a regression model including treatment assignment would significantly reduce the association of HT with ischemic stroke [12]. This was not the case for any marker.

## DISCUSSION

### Interpretation

This nested case-control study was undertaken to elucidate mechanisms underlying the increased stroke risk with HT in the Women's Health Initiative clinical trials, and to attempt to identify women at lower or higher risk for treatment-related stroke. Baseline levels of several biomarkers representing inflammation and coagulation activity, but not the genotypes that were studied here, were associated with increased stroke risk. The finding that women with higher IL-6, PAP, D-dimer, and leukocyte counts at baseline were at lower risk for stroke if assigned to estrogen plus progestin than placebo, but not estrogen alone, is counterintuitive and is likely due to a noncausal association since both hormone treatment groups had similar stroke rates. When one-year changes in biomarkers were examined, only women with treatment-related increases in D-dimer had increased stroke risk with HT. The effect of other risk factors (such as BP, hypertension, and smoking) in the complete Women's Health Initiative cohort has been previously reported [3,4].

The relatively small number of strokes limited our power to definitively detect associations for any lipid parameter. Although low-density lipoprotein (LDL)–cholesterol appears to be a weak risk factor for ischemic stroke in women [13,14], there was evidence in Women's Health Initiative of a trend toward lower stroke risk in women with a reduction in LDL–cholesterol of 0.52–0.73 mmol/l on HT. HDL–cholesterol is more strongly correlated with stroke risk in epidemiologic studies, with protection at levels above 1.45 mmol/l in women aged 45–64 y, and above 1.04 mmol/l in women over age 65 y [13,14]. In this analysis, there was some evidence of a trend toward a lower risk of stroke in women with a greater than 0.16 mmol/l (12%) increase in HDL–cholesterol on HT. Baseline HDL-3, but not HDL-2, appeared to be protective against stroke, perhaps explaining the lack of significance for HDL–cholesterol overall.

Inflammation promotes atherogenic events, including stroke [15,16]. IL-6 is an important regulator of the *CRP* gene [17]. HT with estrogen alone or estrogen plus progestin increases CRP to a similar extent [18]. The rise in CRP with estrogen plus progestin, but not estrogen alone, appears to be IL-6 mediated [19]. These observations suggest that estrogen plus progestin and estrogen alone have differing proinflammatory effects, consistent with the significant interaction between baseline levels of IL-6 and stroke risk that we identified with estrogen plus progestin, but not estrogen alone.

It has been hypothesized that hemostatic activation underlies increased vascular risk with hormones, and it has been previously reported that hormones raise D-dimer levels [20]. Most associations of hemostatic markers and stroke were carried out during acute ischemic stroke, which itself affects the hemostatic system. A prospective study in patients with prior transient ischemic attack or asymptomatic carotid bruits identified prothrombin fragment 1.2, but not PAP or D-dimer, as an independent predictor of subsequent cardiovascular events [21]. In contrast, D-dimer levels independently predicted ischemic stroke in healthy men, whereas prothrombin fragment 1.2 did not [22]. Our findings of higher D-dimer as a risk marker for stroke, and that increases in D-dimer with HT might identify women at risk of stroke, support a role for hemostatic activation in the development of stroke.

### Generalizability

The strengths of this analysis are the ability to prospectively assess the interaction between randomly assigned HT and baseline biomarker levels and changes in biomarker levels. Some limitations should be considered. There were a relatively small number of strokes, confining us to detecting relatively large interactions of treatment with biomarkers. Because we analyzed a large number of biomarkers, some of the results may be due to chance. We studied clinical trial participants who may be different from the general population, so that the results might not be generalizable. A standardized diagnostic approach to stroke was not followed, and this may have led to misclassification of stroke types. Information on stroke subtype was limited. The fact that stroke risk was increased similarly with estrogen alone and estrogen plus progestin, whereas baseline levels of four biomarkers (IL-6, PAP, D-dimer, leukocyte count) appeared to predict a protective association from estrogen plus progestin, raises concerns about the plausibility of these findings. Regarding the findings related to change in D-dimer and stroke susceptibility of women with HT, in view of the number of markers, one interaction might be expected by chance.

### Overall Summary

In this nested case-control study, no biomarker convincingly predicted the stroke risk seen with HT, although some hypotheses were raised. A genome-wide scan is in progress to search for underlying mechanisms, and a proteome study has been proposed to characterize hormone-induced proteins.

## SUPPORTING INFORMATION

CONSORT Checklist Click here for additional data file. (52 KB DOC)

Trial Protocol Click here for additional data file. (147 KB PDF)

## Abbreviations

BMI: body mass index
BP: blood pressure
95% CI: 95% confidence interval
CRP: C-reactive protein
HDL: high-density lipoprotein
HT: hormone therapy
IL-6: interleukin-6
LDL: low-density lipoprotein
Lp(a): lipoprotein (a)
MI: myocardial infarction
MMP-9: matrix metalloproteinase-9
MTHFR: methylene tetrahydrofolate reductase
OR: odds ratio(s)
PAI-1: plasminogen activator inhibitor-1 antigen
PAP: plasmin-antiplasmin complex
TAFI: thrombin activatable fibrinolysis inhibitor, VTE, venous thromboembolism
